# A novel intracerebral hemorrhage-induced rat model of neurogenic voiding dysfunction: Analysis of lower urinary tract function

**DOI:** 10.3892/mmr.2015.3720

**Published:** 2015-05-04

**Authors:** YOUNG-SAM CHO, IL-GYU KO, CHANG-JU KIM, KHAE-HAWN KIM

**Affiliations:** 1 Department of Urology, Kangbuk Samsung Hospital, Sungkyunkwan University School of Medicine, Seoul 110-746, Republic of Korea; 2 Department of Physiology, Kyung Hee University College of Medicine, Seoul 130-701, Republic of Korea; 3 Department of Urology, Gachon University School of Medicine, Gil Medical Center, Incheon 405-760, Republic of Korea

**Keywords:** cerebral hemorrhage, neurogenic urinary bladder, urodynamics, animal models, Fos, nerve growth factor

## Abstract

Neurogenic lower urinary tract dysfunction (NLUTD) is a major problem in patients with various neurological disorders, and may result in debilitating symptoms and serious complications, including chronic renal failure and recurrent urinary tract infections. Clinically, stroke is associated with voiding dysfunction. However, lower urinary tract function evaluation in an intracerebral hemorrhage (ICH) model has not, to the best of our knowledge, been reported. Therefore, in the present study, lower urinary tract function in ICH-induced rats was investigated and the results were compared with those obtained in normal rats. The effects of ICH on peripheral bladder function and central micturition centers [medial preoptic area, ventrolateral gray, pontaine micturition center and spinal cord (lumbar 4 (L4)-L5)] were also examined. Adult female Sprague-Dawley rats were divided into two groups: Control ICH-induced. Induction of ICH in the hippocampal CA1 region was performed using a stereotaxic frame and type IV collagenase. The effects of ICH on the central micturition centers were investigated by simultaneously determining the extent of neuronal activation (c-Fos) and nerve growth factor (NGF) expression, and assessing voiding function (urodynamically using cystometry). The results revealed that induction of ICH significantly enhanced bladder contraction pressure and time, while simultaneously reducing voiding pressure and time. Furthermore, the c-Fos and NGF expression levels in the neuronal voiding centers were significantly increased in the rats with induced ICH as compared with the control rats. Therefore, this ICH-induced NLUTD rat model may be a more appropriate method to analyze NLUTD in stroke patients than a cerebral infarction model, as the former more accurately reflects the nature of the hemorrhage in the two types of stroke.

## Introduction

Neurogenic lower urinary tract dysfunction (NLUTD) is a major problem in patients with various neurological disorders, and may result in debilitating symptoms and serious complications, including chronic renal failure and recurrent urinary tract infections. Important causes of NLUTD include cerebrovascular accident, Parkinson’s disease, spinal cord injury (SCI), multiple sclerosis, diabetes mellitus and peripheral nerve injury due to radical abdominal or pelvic surgery. NLUTD is also common in stroke patients, and the evaluation of bladder function and management of this disorder should be recognized as part of routine rehabilitation (1–3). Occasionally, successful treatment is difficult to quantify due to the irreversibility or progressive nature of strokes. Therefore, neurological investigation of NLUTD in stroke patients is urgently required.

The majority of animal experimental studies of NLUTD have been directed towards the development of models of overactive bladder (OAB) (4). Various animal models of OAB have been analyzed, including a spontaneous hypertensive rat model and a rat model of dopaminergic brain lesions, and various methods have been used to induce OAB, including autoimmune encephalomyelitis, experimental fluid percussion injury to the brain and cerebral infarction using ligation of the common carotid arteries (4). SCI animal models generated by spinal cord compression using electromagnetically controlled watch-maker forceps or spinal cord transection using iridectomy microscissors have also been used to investigate OAB (5,6). Furthermore, bladder outlet obstruction (BOO) models to examine OAB have been generated using a small polyethylene catheter to produce partial urethral obstruction or by intraperitoneal (i.p.) cyclophosphamide (CYP) injection, due to the ease and simplicity of these techniques (7,8). However, these models may not adequately address the characteristics of hemorrhage in stroke patients, although cerebral infarction models are appropriate for ischemic stroke studies. In addition, the relevance of observed changes following SCI, BOO and CYP injection may not reflect conditions in human stroke patients in terms of the possible pathophysiology of NLUTD.

An intracerebral hemorrhage (ICH) model obtained by direct collagenase injection into the brain has been used in numerous neurological studies to evaluate the pathophysiological changes in the brain following ICH and to determine the therapeutic options for treating ICH (9,10). To the best of our knowledge, in the urological literature, no studies that use an ICH model to analyze NLUTD, including OAB and lower urinary tract function, have been reported. Thus, in the present study, lower urinary tract function in ICH-induced rats was investigated and compared with that in normal rats. The effects of ICH on NLUTD with regard to peripheral bladder function and central micturition centers [medial preoptic area (MPA), ventrolateral periaqueductal gray (vlPAG), pontaine micturition center (PMC) and spinal cord (lumbar 4 (L4)-L5)] were also examined.

## Materials and methods

### Experimental animals and treatments

Adult female Sprague-Dawley rats, weighing 260±10 g and aged 10 weeks, were obtained from a commercial breeder (Orient Co., Seoul, Korea). The present study was performed in accordance with the animal care guidelines of the National Institutes of Health and the Korean Academy of Medical Sciences (Seoul, Korea), and was approved by the Kyung Hee University Institutional Animal Care and Use Committee (Seoul, Korea). Each animal was housed under controlled temperature (23±2°C) and lighting (8:00 a.m.–8:00 p.m.) conditions with food and water available *ad libitum* prior and subsequent to experiments. The animals were randomly divided into the following two groups (n=10 in each group): Control and ICH-induced.

### ICH induction

To induce ICH, the rats were anesthetized with Zoletil^®^ 50 (10 mg/kg, i.p.; Vibac Laboratories, Carros, France) and placed in a stereotaxic frame. The needle of a 10 *µ*l Hamilton syringe (Micro 701; Hamilton company, Reno, NV, USA) was inserted through a burr hole into the right hippocampus to the following coordinates: 2.2 mm anterior, 2.2 mm lateral and 4.2 mm depth to bregma. A solution (1 *µ*l) containing 0.2 U collagenase (Type IV; Sigma-Aldrich, St. Louis, MO, USA) was infused over 1 min. The needle was maintained in place for an additional 3 min following the infusion and was subsequently withdrawn slowly. The animals in the sham-operation group received 1 *µ*l physiological saline by the same method.

### Cystometry

Rat bladder function was evaluated by cystometry 14 days after the ICH surgery. The rats were anesthetized with 10 mg/kg Zoletil® 50 (i.p.). A sterile polyethylene catheter (PE50) was inserted into the urethra through the bladder dome. The catheter was connected to a pressure transducer (Harvard Apparatus, Holliston, MA, USA) and syringe pump (Harvard Apparatus) via a three-way stopcock to record the intravesical pressure. When the bladder had emptied, cystometry was conducted by infusing 0.5 ml saline into the bladder. Bladder contraction pressure and time were monitored using Labscribe (iWork System Inc., Dover, NH, USA).

### Preparation of tissues

The rats were sacrificed immediately following cystometric determination of the contraction pressure and time in the bladder. Briefly, the animals were anesthetized with Zoletil 50^®^ (10 mg/kg, i.p.; Vibac Laboratories), and were transcardially perfused with 50 *µ*m phosphate-buffered saline (PBS), followed by 4% paraformaldehyde in 100 mm sodium phosphate buffer at pH 7.4. The brain was removed, postfixed in the same fixative overnight and transferred to a 30% sucrose solution for cryoprotection. Subsequently, 40 *µ*m serial coronal sections were produced with a freezing microtome (Leica Biosystems Nussloch GmbH, Nussloch, Germany). The PMC was defined as the region spanning Bregma −9.68 to −9.80 mm, the vlPAG as the region spanning Bregma −7.64 to −8.00 mm, the MPA as the region spanning Bregma −0.26 to 0.80 mm and the spinal cord as the L4–L5 region. An average of 10 sections were collected from each rat for each region and were used for immunohistochemical analysis.

### Immunohistochemistry for c-Fos and nerve growth factor (NGF)

Rabbit polyclonal anti-c-Fos (cat. no. sc-52) or mouse monoclonal anti-NGF (cat. no. sc-365944) antibodies (Santa Cruz Biotechnology, Inc., Santa Cruz, CA, USA) at 1:1,000 dilutions were incubated overnight with free-floating tissue sections at 4°C. The sections were washed three times with PBS for 3 min, and were subsequently incubated for 1 h at room temperature with biotinylated anti-rabbit (cat. no. BA1000) or anti-mouse (cat. no. BA2000) secondary antibodies (1:200 dilution; Vector Laboratories, Burlingame, CA, USA) as appropriate. After washing three times with PBS for 3 min, the sections were incubated for 1 h at room temperature with avidin-biotin-peroxidase complex (Vector Laboratories). The sections were washed a further three times with PBS, and were then incubated in a solution consisting of 0.05% 3,3′-diaminobenzidine (Sigma-Aldrich) and 0.01% H_2_O_2_ in 50 mm Tris-buffered saline (pH 7.6) for ~3 min to visualize immunoreactivity. The sections were mounted onto gelatine-coated slides subsequent to washing three times with PBS. The slides were air-dried at room temperature overnight and coverslips were mounted using Permount™ Mounting Medium (Fisher Scientific, Waltham, MA, USA).

### Data analysis and statistics

The numbers of c-Fos-positive and NGF-positive cells in the neuronal voiding centers [MPA, vlPAG, PMC and spinal cord (L4–L5)] were counted hemilaterally through a light microscope (BX-51; Olympus Corporation, Tokyo, Japan). The area of the neuronal voiding center in each slice was measured using the Image-Pro^®^ Plus computer-assisted image analysis system (Media Cyberbetics Inc., Silver Spring, MD, USA) attached to the light microscope (BX-51; Olympus Corporation). The data were analyzed using SPSS software (ver. 20.2; IBM, Armonk, NY, USA) and are expressed as the mean ± standard error of the mean. The results of the sham surgery and ICH-induced groups were compared using an independent paired Student’s t-test. P<0.05 was considered to indicate a statistically significant difference.

## Results

### Effect of ICH on urodynamic parameters

**Figure 1:**
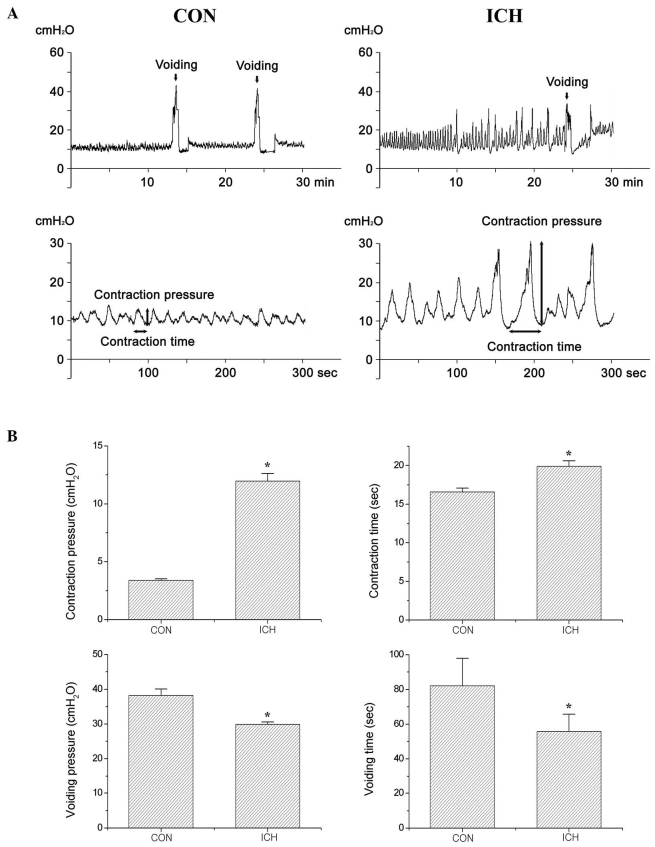
Effect of induction of ICH on urodynamic parameters. (A) Cystometry results for each group. (B) Analysis of bladder contraction (pressure/time) and voiding function (pressure/time). Results are presented as the mean ± standard error of the mean. ^*^P<0.05 vs. the control group. ICH, intracerebral hemorrhage; CON, control group.

The bladder contraction and voiding parameters (pressure and time) obtained by cystometry are presented in Fig. 1 and Table I. These results indicate that bladder contraction pressure (P<0.05) and time (P<0.05) were significantly increased by the induction of ICH as compared with the control treatment. By contrast, the voiding pressure (P<0.05) and time (P<0.05) were significantly reduced in the rats with ICH induction compared with the control rats.

### Effect of ICH induction on c-Fos expression in the neuronal voiding centers

**Figure 2:**
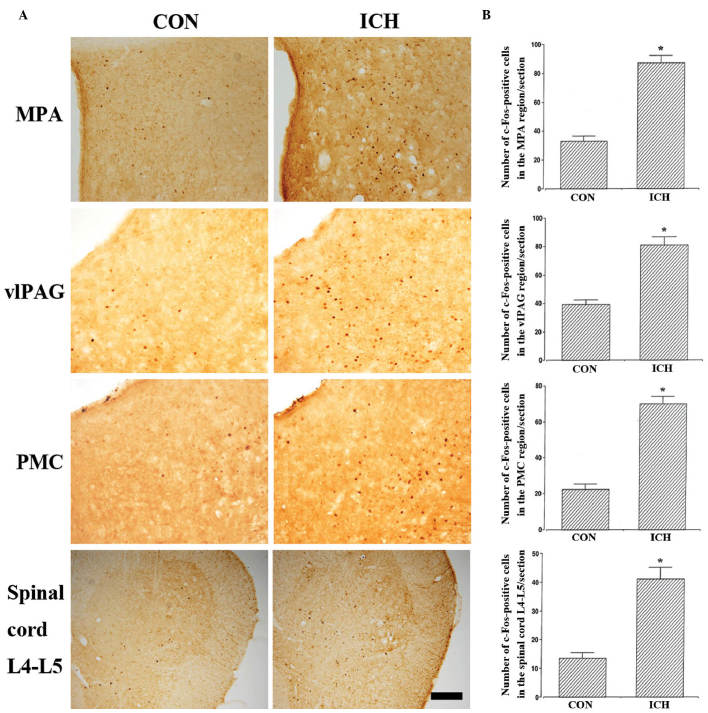
Effects of ICH induction on c-Fos expression in the neuronal voiding centers. (A) Photomicrographs of c-Fos-positive cells in the neuronal voiding centers. The sections were stained for c-Fos (brown staining). Scale bar, 200 *µ*m. (B) Number of c-Fos-positive cells in each group. The results are presented as the mean ± standard error of the mean. ^*^P<0.05 vs. the control group. ICH, intracerebral hemorrhage; CON, control group; MPA, medial preoptic area; vlPAG, ventrolateral gray; PMC, pontaine micturition center; L, lumbar.

The results of the analysis of c-Fos-positive cells in the neuronal voiding centers are presented in Fig. 2 and Table I. These results indicate that the c-Fos expression levels in the neuronal voiding centers were significantly increased in the rats with induction of ICH compared with the control rats (P<0.05).

### Effects of ICH induction on NGF expression in the neuronal voiding centers

**Figure 3:**
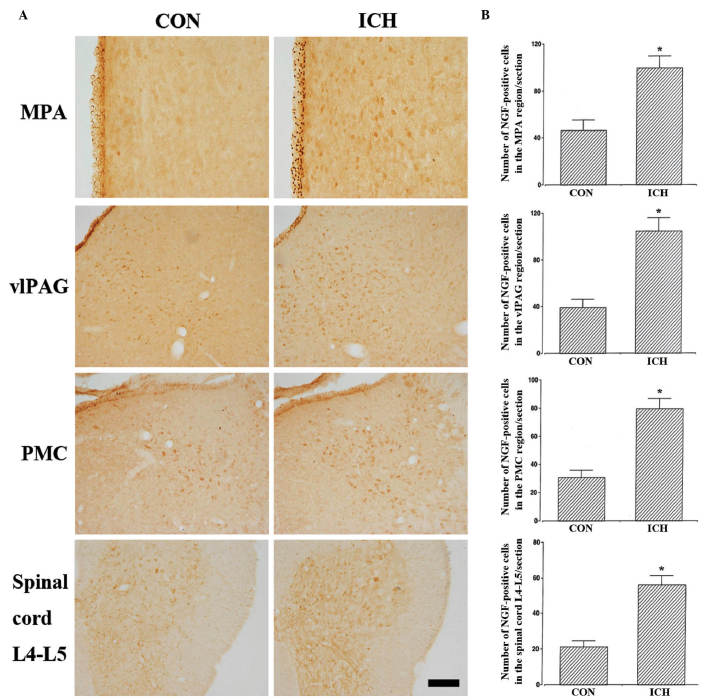
Effect of ICH induction on NGF expression in the neuronal voiding centers. (A) Photomicrographs of c-Fos-positive cells in the neuronal voiding centers. The sections were stained for c-Fos (brown staining). Scale bar, 200 *µ*m. (B) Number of NGF-positive cells in each group. Results are presented as the mean ± standard error of the mean. ^*^P<0.05 vs. the control group. ICH, intracerebral hemorrhage; NGF, nerve growth factor; CON, control group; MPA, medial preoptic area; vlPAG, ventrolateral gray; PMC, pontaine micturition center; L, lumbar.

The results from the analysis of NGF-positive cells in the neuronal voiding centers are presented in Fig. 3 and Table I. These results indicate that NGF expression levels in the neuronal voiding centers were significantly increased in the rats with induction of ICH compared with the control rats (P<0.05).

## Discussion

The terms ICH and hemorrhagic stroke are used interchangeably and epidemiological studies indicate that 8–18% of strokes result from hemorrhagic insult (11). In ICH, bleeding occurs directly into the brain parenchyma. The predominant mechanism is considered to be leakage from small intracerebral arteries damaged by chronic hypertension (11). Furthermore, in 20–40% of patients with ischemic infarction, hemorrhagic transformation may occur within one week of ictus (12). Therefore, hemorrhage is an important pathophysiological factor in hemorrhagic and ischemic stroke.

Urinary symptoms are associated with disability and are reported to exert a considerable impact on the quality of life of stroke survivors (13). Urinary incontinence (UI) has been reported in 47% of stroke patients in the acute phase and in 19% of patients at the six-month follow-up (2). Urinary retention has been reported in 47% of stroke patients within 72 h of the cerebrovascular accident (1) and in 29% within four weeks of the stroke (3). Urinary symptoms, including alterations in urinary frequency, nocturia and a significantly higher rate of urinary tract infections, are observed in patients with urinary retention or incomplete bladder emptying (3). In a previous study, the frequencies of urine storage and emptying disorders in hemorrhagic and ischemic stroke patients with persistent bladder dysfunction were similar: 73.3% in the hemorrhagic stroke group versus 63.6% in the ischemic stroke group (14).

However, the majority of experimental studies of the pathophysiology and treatment of NLUTD in stroke have used a cerebral infarction animal model (4). This experimental model, however, does not accurately reflect hemorrhagic stroke. Therefore, this is not an optimal animal model in which to analyze NLUTD associated with hemorrhagic stroke. The induction of ICH by direct collagenase injection into the brain has been used in previous neurological studies (9,10). In the present study, previously reported procedures for ICH induction in the hippocampal CA1 region using a stereotaxic frame and type IV collagenase were adopted, and ICH-induced changes in bladder function and neuronal voiding centers were investigated.

Bladder contraction pressure and time were found to be significantly increased in ICH-induced rats compared with normal rats, whereas the voiding pressure and time were significantly reduced by the induction of ICH, which indicated that ICH resulted in a deterioration in bladder function and as a result, the induction of OAB. Furthermore, the reductions observed in voiding pressure and time in the ICH-induced rat model are consistent with the findings in various previous OAB models (6–8), demonstrating that OAB was induced in the ICH-induced rat model in the present study. The findings from the present study provide further support for those from a previous study that reported a significant reduction in bladder capacity 2 h after middle cerebral artery (MCA) occlusion in rats (15). Furthermore, the mean micturition threshold pressure increased significantly in the cerebral infarction group in that previous study, but the bladder contraction pressure was not significantly affected by left MCA occlusion compared with a sham group (15). In another study, cerebral infarction induced by MCA occlusion reduced bladder capacity in rats, but did not produce a change in bladder contraction pressure (16). The discrepancy in bladder contraction results between the present study and previous studies may be due to differences in the stroke mechanism, namely hemorrhage versus ischemia. This highlights the importance of the method used to induce stroke in experimental animal NLUTD models.

The PMC is important in the control of urinary bladder function. The hypothalamic PAG and MPA regions have been associated with the PMC (17). The PAG-PMC projection is hypothesized to be involved in the micturition reflex. The vlPAG functions as a central sensorimotor integrative relay of the micturition reflex via the reception of sensory information concerning bladder fullness and this area directly projects to the PMC (18). Neurons in the PAG regulate the micturition reflex in animals and humans, since lesions in the PAG cause severe urinary dysfunction (18,19). In addition, the PMC is densely innervated by the MPA (18). The MPA sends projections to the PMC that synapse on neurons directly through projections to the spinal cord (17).

c-Fos expression occasionally serves as a marker for stimuli-induced changes in the metabolic activity of neurons under various conditions (20). In a previous study, stimulation of lower urinary tract symptoms (LUTS) was shown to cause changes in neuronal activity in neuronal micturition centers (21). Increased NGF levels have been observed in the urine of patients with interstitial cystitis and painful bladder contractions (22). NGF overexpression in the bladder and urethra is associated with modulation disability of micturition in patients with UI (23,24). One biochemical study demonstrated that NGF regulates afferent bladder neuronal plasticity subsequent to partial urethral obstruction in female rats (24). Increased levels of NGF in the bladder, spinal cord and dorsal root ganglia have been associated with bladder hyperreflexia following SCI in rats (25).

In the present study, the expression levels of c-Fos and NGF in the neuronal voiding centers was significantly increased following the induction of ICH, indicating that ICH facilities bladder instability through enhanced neuronal activation in central micturition areas. These findings are consistent with those of a previous study that reported increased NGF levels in the bladder tissues and urine of patients with OAB and detrusor overactivity (26), and increased c-Fos expression levels in the neuronal voiding centers in OAB model rats (27). Understanding the underlying mechanisms by which ICH affects urinary function is important. The limbic system is a complex set of neurological structures that lies on the two sides of the thalamus immediately under the cerebrum, and includes the hypothalamus, hippocampus, amygdala and a number of proximal areas (28). These limbic systems are known modulators of urinary function. In particular, the hypothalamus has monosynaptic connections with the PAG and PMC (28), and modulates the bladder reflex and therefore control of urinary function. Animal studies have shown connections between the thalamus and the prefrontal regions and also the PAG, which indicates that this structure is important in the relay of information, presumably including bladder sensation (21). Matsuura *et al* (29) noted that the thalamus was activated during the ‘full bladder’ state. Therefore, hemorrhagic attacks of the limbic system result in changes in the synapses of limbic areas, including the hypothalamus and hippocampus, and neuronal activity alteration in the neuronal voiding areas. NLUTD, including OAB and UI, may result from these alterations.

The ICH-induced NLUTD rat model in the present study may therefore be a more appropriate model to analyze NLUTD in stroke patients than the cerebral infarction model, as the model in the present study more accurately reflects the nature of the hemorrhage in hemorrhagic and ischemic stroke. Little consensus has been reached with regard to how stroke patients with NLUTD should be treated or monitored, and current treatment options for NLUTD are also limited. Animal studies are required for the examination of the efficacy or toxicity of novel medications. In the present study, ICH-induced expression of c-Fos and NGF in the neuronal voiding centers was observed in an animal model of NLUTD. Enhanced c-Fos and NGF expression levels in the voiding centers indicates activated neuronal systems that presumably induce LUTS associated with NLUTD. Thus, the animal model developed in the present study may be useful for future investigations of NLUTD associated with stroke, particularly hemorrhagic stroke.

## Figures and Tables

**Table I:** Changes in urodynamic parameters, c-Fos and NGF expression following the induction of ICH.

Variable	Control	ICH
Bladder contraction parameter		
Pressure (cm H_2_O)	3.30±0.13	11.97±0.64^a^
Time (sec)	16.58±0.50	19.89±0.70^a^
Voiding parameter		
Pressure (cm H_2_O)	38.23±1.86	29.93±1.50^a^
Time (sec)	82.00±15.90	55.78±10.00^a^
c-Fos expression (no. cells/section)		
MPA	33.22±3.11	87.20±5.19^a^
vlPAG	39.50±2.88	81.20±5.69^a^
PMC	22.44±3.09	70.10±4.01^a^
L4–L5	13.55±1.80	41.09±4.11^a^
NGF expression (no. cells/section)		
MPA	46.38±9.01	99.44±10.19^a^
vlPAG	39.14±6.90	104.45±11.39^a^
PMC	30.61±5.10	79.50±7.20^a^
L4–L5	21.45±3.10	56.11±5.10^a^

Data are presented as the mean ± standard error of the mean. N=10 in each group.

a P<0.05 vs. the control group. NGF, nerve growth factor; ICH, intracerebral hemorrhage; MPA, medial preoptic area; vlPAG, ventrolateral periaqueductal gray; PMC, pontaine micturition center; L, lumbar spinal cord.
